# Predictors of knowledge and practice of exclusive breastfeeding among health workers in Mwanza city, northwest Tanzania

**DOI:** 10.1186/s12912-016-0192-0

**Published:** 2016-12-30

**Authors:** Lucy E. Chale, Tanis R. Fenton, Neema Kayange

**Affiliations:** 1 Bugando Medical Centre, Department of community health services, Faculty of Nursing, Catholic University of Health and allied sciences (CUHAS), Mwanza, Northwest Tanzania; 2 Nutrition Services, Alberta Health Services, Department of Community Health Sciences, Alberta Children’s Hospital Research Institute, O’Brien Institute for Public Health, Faculty of Medicine, University of Calgary, Calgary, AB Canada; 3 Bugando Medical centre, Department of Paediatrics, Faculty of Medicine, Catholic University of Health and Allied Sciences (CUHAS), Mwanza, Northwest Tanzania

**Keywords:** Health workers, Exclusive breastfeeding, Knowledge, Practices

## Abstract

**Background:**

Universal exclusive breastfeeding (EBF) for the first 6 months is estimated to reduce infant mortality by 13–15% (9 million) in resource poor countries. Although 97% of women initiate breastfeeding in Tanzania, exclusive breastfeeding for 6 months remains below 50%. Accurate knowledge and practical skills pertaining to exclusive breastfeeding among health workers is likely to improve breastfeeding rates. Our study reports the health workers’ knowledge and practice on EBF in Mwanza City, northwest of Tanzania.

**Methods:**

One principal researcher and two research assistants conducted data collection from 11 June–6 July 2012. In total, 220 health care workers including: 64 clinicians (medical specialists, residents, registrars, assistant medical officers and clinical officers) and 156 nurses were interviewed using a structured knowledge questionnaire. Amongst 220 health workers, 106 were observed supporting Breastfeeding using a checklist. Logistic regression was used to determine factors associated with exclusive breastfeeding knowledge and desirable skills.

**Results:**

Almost half of the 220 health workers interviewed correctly described EBF as defined by the World Health Organization. Only 52 of 220 respondents had good knowledge. In the adjusted analysis, working at hospital facility level compared to dispensary (OR 2.1; 95% CI 1.1–4.0, *p*-value = 0.032) and attending on job training (OR 2.7; 95% CI 1.2–6.1, *p*-value = 0.015) were associated with better knowledge. In total, 38% of respondents had a desirable level of practical skills. Clinicians were more likely to have good practice (OR 3.6; 95% CI 1.2–10.8; *p*-value = 0.020) than nurses. Most of the health workers had no training on EBF, and were not familiar with breastfeeding policy.

**Conclusion:**

Less than 25% of healthcare workers surveyed had good knowledge of EBF. These findings identify the need for comprehensive training and mentoring of health workers on exclusive breastfeeding, making breastfeeding policies available and understood, along with supportive supervision and monitoring.

**Electronic supplementary material:**

The online version of this article (doi:10.1186/s12912-016-0192-0) contains supplementary material, which is available to authorized users.

## Background

Breastfeeding is the process of feeding the infant with mother’s milk, either by direct nipple-baby mouth contact or by expressed breast milk. Exclusive breastfeeding (EBF) is the practice of feeding the infant breastmilk only for the first 6 months of life without any other type of food or drink, not even water [1]. In 1990, the WHO and UNICEF jointly adopted the Innocent Declaration on the protection, promotion and support of breastfeeding, and emphasized the importance of EBF [2]. The declaration urges all governments to develop national breastfeeding policies and set appropriate national targets. One of the key deliberations, as far EBF is concerned, was to impart the health workers and staff in all sections of health services delivery adequate knowledge and skills to support breastfeeding [2].

The benefits of breastfeeding are numerous: not only is it considered complete nutrition for the first 6 months of life, exclusive breastfeeding is associated with preventing life-threatening infections in infants, as well as health benefits for mothers [3, 4]. It has been estimated that at 90% EBF, death of children less than 5 years due to respiratory tract infections, diarrhoea diseases and neonatal sepsis could be prevented [5, 6]. Breastmilk contains immune cells and immunoglobulins from the mother that have a documented protective effect on infants from infections [7]. Specifically, the immune cells are macrophages and the neutrophils that can destroy harmful bacteria; and the immunoglobins, which help to protect infants’ mucosal surfaces against entry of pathogenic bacteria and viruses [7, 8]. These immune substances could prevent up to 13–15% [9 million] deaths of children under 5 years in resource poor settings [9]. Other EBF benefits include: aiding in uterine contraction through the release of oxytocin; suppress ovulation; and increased bonding between the mother and the newborn [9]. Even in the areas where HIV prevalence is high, especially in sub-Saharan Africa, EBF has been associated with lower rates of mother to child HIV transmission [10, 11].

In Tanzania, approximately 97% of infants are breast-fed at some point in 2010, up from 41% in 2005 [12]. However, only about one half of women practice EBF up to 6 months. The rates of EBF fall off rapidly with infant’s age: < 2 months (81%), 2–3 months (33%) and 4–5 months (36%) [13].

The global strategy for Infant and Young Child feeding emphasizes the need for health workers to be trained in counselling and assistance skills for breastfeeding and complementary feeding; breastfeeding and HIV; feeding during illnesses; and health worker’s role in implementing international code conduct of marketing milk-substitutes [14]. Health workers are responsible for supporting women to EBF at the health facilities and in the community [15–17]. A study conducted by the Tanzania Food and Nutrition Centre in Kagera, Mbeya and Kilimanjaro, revealed a large knowledge gap in terms of the recommended duration of EFB among Health Service Providers (HSP) as only 26.5% could recall the 4–6 month EBF recommendation [18]. Although 70% of breastfeeding mothers confirmed receiving information from health workers, 13% of the health workers were not able to demonstrate pertinent breastfeeding skills such baby positioning and attachment [18]. These substantial knowledge and skill gaps put breastfeeding mothers at risk of receiving incorrect information from poorly informed health providers, which likely contributes to the low prevalence of EBF among women [19]. The World Health Organization recommend in their 2nd step of Ten Steps To Successful Breastfeeding that health workers be trained on EBF for at least 18 h plus 3 h of on the job training [1].

Our study reports factors associated with knowledge and practice of health workers working in one of rapidly expanding cities in Tanzania.

## Methods

The study was cross-sectional and descriptive, conducted among health workers in the study area. The study had two parts: face-to-face interviews with health workers, and observing health worker practical skills using a checklist.

The study was conducted in Nyamagana and Ilemela Districts, in the City of Mwanza, Tanzania. Nyamagana district total population was 210,735 whereas Ilemela district had 265,911 people [20, 21]. The health workers in the maternal, post-natal, newborn and child health clinics were recruited in the study because are routinely involved in supporting breastfeeding. The total number of health workers working in the maternity, postnatal wards and child health clinics in the two districts, clinicians such as medical specialists, residents, registrars, assistant medical officers and clinical officers were 220 and the nurses such as registered, enrolled nurses and auxiliary nurses were 644 (Table 1).

**Table 1:** Sampling framework of health workers in Nyamagana and Ilemela districts

Health facilities	Total clinicians^a^	Total nurses^b^	Clinicians sampled (interviewed)^c^	Nurses sampled (interviewed)^d^
Bugando Medical Centre	144	327	36 (34)	70 (60)
Sekou Toure Regional Hospital	20	172	5 (4)	35 (28)
Nyamagana District Hospital	13	30	8 (8)	18 (14)
Buzuruga Health Centre	11	15	5 (5)	15 (9)
Karume Health Centre	7	11	5 (5)	14 (11)
Igoma Health Centre	6	18	3 (2)	8 (6)
Makongoro Health Centre	5	15	2 (2)	22 (18)
Nyakato Dispensary	5	11	2 (1)	4 (3)
Pasiasi Dispensary	2	5	2 (2)	5 (3)
Nyerere Dispensary	5	15	2 (1)	4 (2)
Buhongwa Dispensary	2	5	1 (0)	5 (2)
Total	220	624	71 (64)	200 (156)

^a^The total number of clinicians (medical specialists, residence, registrars, assistant medical officers and clincial officers) at 11 health facilities; ^b^total number of nurses at the health facilties, which includes registered, enrolled and auxillary nurses; ^c^number of interviewed clinicians (% of the interviwed, ^a^demoninator); number of interviwed nurses interviwed (% of the interviwed, ^b^demoninator)

Eleven health facilities out of 30 in the Nyamagana and Ilemela districts were purposively selected to include: seven urban and four rural. There were two consultant and referral hospitals, one district hospital, four health centres and four dispensaries included in the study. The consultant, referral hospitals and district hospital are capable of providing emergency comprehensive obstetrics and neonatal care, including supporting emergency feeding complications. In contrast, the services provided at the health centres and the dispensaries are basic or routine maternal and newborn services. We sampled proportionate to size to allocate the sample by health cadre and by facility (Table 1). Overall, 220 health care workers: 64 clinicians and 156 nurses responded to the structured knowledge questionnaire. Each of these study participants were adults and each signed a consent form to participate in the study as required by the Joint Ethical Committee of CUHAS and Bugando Medical Centre. Once the number of possible respondents for every facility was determined, all eligible individuals available at the workplace and willing to participate were recruited into the study and interviewed until the desired number was attained at that health facility. Amongst 220 health workers who responded to the questionnaire, 110 (50%) were randomly sampled for the breastfeeding practical observations checklist. Four individuals declined to undergo the practical session, hence 106 observations were performed.

Two data collection tools were used. First, a structured questionnaire, developed based on EBF technical references [22, 23] and study objectives, was used to collect knowledge and attitude data from the health workers, as well as socio-demographic characteristics (Additional file 1). The researchers asked each respondent 17 knowledge questions. The response for each question was ranked using a 1–4 Likert scale such as: incorrect response (1); partially correct (2); mostly correct response (3); and correct (4). The total knowledge score per respondent was later categorized into two groups, desirable and undesirable as: < 8/17 (47%) total score was considered undesirable and above 47% as desirable. Second, a breastfeeding observation checklist to observe breastfeeding practical skills was adapted from WHO/UNICEF Baby Friendly Hospital Initiative guidelines [24]. All tools were in English.

Only the principal researcher administered the 23 item observation checklist to observe the health workers helping the breastfeeding mother. Each observation criterion was give one mark. At the end of each observation, the marks were added and calculated as percentage of the total expected score. Scores were later grouped into two categories: if the interaction scored 12 or less out of 23 (<55%), it was categorized as undesirable. A score of 13 and above was considered desirable.

The data were coded and entered into SPSS for statistical analysis. We used univariate analysis followed by multivariate logistic regression to determine the factors associated with knowledge and with practical skills of health workers. The factors considered in the regression modelling included: type of health facility, age, sex, cadre, on job training and work longevity. Odds ratios with 95% confidence intervals were calculated and factors with *p*-value less than 0.05 were considered statistically significant.

## Results

### Demographic characteristics of health workers

In total, 220 (81%) of the sampled respondents were interviewed, which is 64 (90%) of the clinicians and 156 (78%) of the nurses of the targeted sample. The respondents’ ages varied from 23 to 58 years. The mean age was 37.7 years (SD 8.8). Most of the participants, 132 (60%) had more than 4 years of working experience after professional training (Table 2). The majority of health workers, 183 (83.2%) reported no on-the-job training on EBF after obtaining their professional qualifications. Among those who had on-the-job training, 57% had only 1–3 weeks of training (Table 2).

**Table 2:** Demographic characteristics of the health workers of Nyamagana and Ilemela districts of Mwanza city included in the study

Variables	Nyamagana *n* (%)	Ilemela district *n* (%)	Total *n* (%)
Sex of the study group (*n* = 220)^a^			
Male	25 (11.4%)	14 (6.3%)	39 (17.7%)
Female	121 (55%)	60 (27.3%)	181 (82.3%)
Age of the study group (*n* = 220)^a^			
19–30 years	26 (11.8%)	16 (7.3%)	42 (19.1%)
31–40 years	67 (34.4%)	29 (13.2%)	96 (43.6%)
41–50 years	35 (15.9%)	18 (8.2%)	53 (24.1%)
51 years and above	18 (8.2%)	11 (5%)	29 (13.2%)
Health workers cadres (*n* = 220)^a^			
Registered nurse	54 (24.5%)	27 (12.3%)	81 (36.8%)
Enrolled nurse	40 (18.2%)	25 (11.3%)	65 (29.5%)
Doctor	36 (16.4%)	4 (1.8%)	40 (18.2%)
Clinician (CO and AMO)	10 (4.5%)	14 (6.4%)	24 (10.9%)
Auxiliary Nurse	6 (2.7%)	4 (1.8%)	10 (4.5%)
Years after professional training (*n* = 220)^a^			
less than 1 year	12 (5.5%)	8 (3.6%)	20 (9.1%)
1 to 3 years	46 (20.9%)	22 (10%)	68 (30.9%)
4 to 8 years	42 (19.1%)	16 (7.3%)	58 (26.4%)
9 years and more	46 (20.9%)	28 (12.7%)	74 (33.6%)
Ever had training on exclusive breastfeeding (*n* = 220)^b^			
Yes	26 (11.8%)	11 (5%)	37 (16.8%)
No	120 (54.6%)	63 (28.6%)	183 (83.2%)
Length of exclusive breastfeeding training (*n* = 37)^c^			
less than 1 week	12 (32.4%)	3 (8.1%)	15 (40.5%)
1 to 3 weeks	13 (35.2%)	8 (21.6%)	21 (56.8%)
4 to 6 weeks	1 (2.7%)	0 (0%)	1 (2.7%)

^a^The demographic data of health workers (*N* = 220), which include sex, age and cadre and number of years after professional training; ^b^number trained/not trained on exclusive breastfeeding (*N* = 220), amongst ^c^(*N* = 37) were ever trained on exclusive breastfeeding: the length of training ranged from less than a week to 4–6 weeks

### Breastfeeding policy training

All participants were asked about the availability of a breastfeeding policy in their facility, if the policy was visibly posted and about their familiarity with the policy. The majority of health workers, 120 (54.5%) said the health facility had no breastfeeding policy. However, of those who did report existence of a breastfeeding policy, only 15 (25.4%) (6.8% of the whole sample) stated the policy was displayed at the health facility. Similarly, most of the health workers, 149 (67.7%) reported not being familiar with the national breastfeeding policy.

In response to the questions about breastfeeding policy training, 17 (7.7%) reported having had any on-the-job training on breastfeeding policy, of whom only 5 (29%) of these had the recommended length of cumulative training of not less than 18 h [1] (Table 3). Among the 17 health workers interviewed who received training, most (82%) received training that included the 10 steps of EBF. A minority of the health workers, 23 (10%) reported that health facilities donated formula to babies within the year prior to the study.

**Table 3:** Health workers reported training on breastfeeding policy and counselling

Variables	Number	Percent
Trained on breastfeeding Policy (*n* = 220)^a^		
Yes	17	7.7
No	203	92.3
Length of trained on breastfeeding Policy (*n* = 17)^a^		
Less than 5 h	7	41.2
5 to 10 h	3	17.6
11 to 18 h	2	11.8
19 h or more	5	29.4
Training covering 10 steps to successful breastfeeding (*n* = 17)^a^		
Yes	14	82.4
No	3	17.6
Cumulative Hours of Mentoring after training (*n* = 17)^a^		
Yes, for 30 min to 1 h	5	29.4
Yes, for 1 h to 2 h	4	23.5
Yes, for 3 h and more	2	11.8
Not at all	6	35.3
Ever trained on Breastfeeding counseling (*n* = 220)^b^		
Yes, during pre/in-service training	43	19.5
Yes during seminar and workshop	44	20
Not at all	133	60.5
Milk formulae donations to babies over the past 1 year (*n* = 220)^c^		
Yes	23	10.5
No	121	55
Don’t know	76	34.5

^a^The number of health workers (*N* = 220), amongst them, 17 (7.7%) were trained on breastfeeding policy: subsequently segregated according to the length of training, training covering ten steps and total number of hours mentored after training; ^b^number of health workers according to trained on breastfeeding counselling; ^c^health workers knowledge on milk formulae donation to the health facility

### Factors associated with knowledge of exclusive breastfeeding among health workers

Overall, about one half of respondents, 114 (52%), provided desirable responses to the 17 questions asked about EBF knowledge, whereas 106 (48%) knowledge scores were considered undesirable (Table 4). On the other hand, 153 (69.5%) of health workers thought “crying a lot” was justification for complementary feeds before the age of 4 months. Almost half, 117 (53%) of health workers interviewed gave an incorrect description of the definition of Exclusive Breastfeeding.

**Table 4:** Univariate and multivariate factors associated with exclusive breastfeeding knowledge among the health workers

Variables (*n* = 220)	Level of knowledge^a^		Univariate analysis		Multivariate analysis^b^	
	Desirable (*n*, %)	Undesirable (*n*, %)	OR [95% CI]	*p*-value	OR [95% CI]	*p*-value
Sex						
Male	20 (51.3)	19 (48.7)	1			
Female	94 (51.9)	87 (48.1)	1.0 [0.5–2.1]	0.941	1.1 [0.5–2.7]	0.784
Age						
> 40 years	40 (46.5)	46 (53.5)	1			
≤ 40 years	74 (55.2)	60 (44.8)	1.4 [0.8–2.4]	0.208	1.4 [0.8–2.6]	0.253
Hospital level						
Disp/HC	26 (36.1)	46 (63.9)	1			
Hospital	88 (59.5)	60 (40.5)	2.6 [1.4–4.6]	0.001	2.1 [1.1–4.0]	0.032
Cadre (*n* = 220)						
Nurses	83 (53.2)	73 (46.8)	1			
CO/AMO	7 (29.2)	17 (70.8)	0.4 [0.1–0.9]	0.033	0.4 [0.1–1.2]	0.093
Doctors	24 (60.0)	16 (40.0)	1.3 [0.7–2.7]	0.442	0.9 [0.4–2.2]	0.849
Years since profession training						
> 3 years	62 (47.0)	70 (53.0)	1			
≤ 3 years	52 (59.1)	36 (40.9)	1.6 [0.9–2.8]	0.079	1.8 [1.0–3.3]	0.062
Job Training (*n* = 220)						
No	88 (48.1)	95 (51.9)	1			
Yes	26 (70.3)	11 (29.7)	2.6 [1.2–5.5]	0.016	2.7 [1.2–6.1]	0.015

^a^Desirable knowledge was determines by scoring 8 or more of the 17 knowledge questions, whereas undesirable was scoring than 8 questions. ^b^Variables controlled were: sex, age, facility level, cadre, and years since professional training and breastfeeding on the job training. Multivariate analysis age, sex and health facility levels were controlled as potential confounders

Univariate logistic regression analysis indicated that working at the hospital was associated with better knowledge on exclusive breastfeeding compared to a dispensary (OR 2.6; 95% CI 1.4–4.6, *p*-value = 0.001) (Table 4). Attending on-the-job training was significantly associated with desirable knowledge, (OR 2.6; 95% CI 1.2–5.5, *p*-value = 0.016). Of importance, clinical officers and assistant medical officers were less likely to have desirable knowledge than nurses (OR 0.4; 95% CI 0.1–0.9, *p*-value = 0.033). On multivariate logistic regression analysis, once sex, age, facility level, cadre, and years since professional training and on the job breastfeeding training were controlled for, similar factors as for univariate were associated with desirable knowledge on breastfeeding - working at hospital facility level compared to dispensary (OR 2.1; 95% CI 1.1–4.0, *p*-value = 0.032) and attending on-the-job training (OR 2.7; 95% CI 1.2–6.1, *p*-value = 0.015) were associated with better knowledge. Job cadre was no longer significantly associated with desirable EBF knowledge in the multivariate analysis.

### Factors associated with the desirable exclusive breastfeeding practices among health workers

Among 220 health workers who participated in the study, almost half 106 (48.1%) were observed using step-by-step checklist of 23 observations to assist the mother with breastfeeding her baby. Among those we observed, the majority, 66 (62%) had undesirable practical skills and compared to 40 (38%) who exhibited desirable practical skills (Table 5).

**Table 5:** Univariate and multivariate factors associated with desirable exclusive breastfeeding practices of the health workers

Participants’ variable (*n* = 106)	Demonstrating breastfeeding^a^		Univariate analysis		Multivariate analysis^b^	
	Desirable (*n*, %)	Undesirable (*n*, %)	OR [95% CI]	*p*-value	OR [95% CI]	*p*-value
Sex						
Male	8 (52.1)	6 (42.9)	1			
Female	32 (34.8)	60 (65.2)	0.4 [0.1–1.3]	0.116	0.7 [0.2–2.9]	0.649
Age						
> 40 years	20 (33.3)	40 (66.7)	1			
≤ 40 years	20 (43.5)	26 (56.5)	1.7 [0.8–3.8]	0.195	1.1 [0.4–2.8]	0.84
Hospital level						
Disp/HC	19 (48.7)	20 (51.3)	1			
Hospital	21 (31.3)	46 (68.7)	0.5 [0.2–1.1]	0.077	0.4 [0.2–1.1]	0.084
Cadre (*n* = 106)						
Nurses	24 (29.6)	57 (70.4)	1			
Clinicians	16 (64.0)	9 (36.0)	4.2 [1.6–10.9]	0.003	3.6 [1.2–10.8]	0.020
Years since profession training						
> 3 years	20 (33.3)	40 (66.7)	1			
≤ 3 years	20 (43.5)	26 (56.5)	1.5 [0.7–3.4]	0.287	1.4 [0.6–3.4]	0.477
Job Training						
No	33 (37.1)	56 (62.9)	1			
Yes	7 (41.2)	10 (58.8)	1.2 [0.4–3.4]	0.75	2.7 [0.5–6.0]	0.339

^a^desirable practical skills was determines by the health workers scoring 51 or more grade on the likert scale of 23 items checklist, whereas undesirable was scorimng less than 51 grades. ^b^Variables controlled were: sex, age, facility level, cadre, and years since professional training and breastfeeding on the job training. Multivariate analysis was controlled for age and sex as potential confounders

On univariate logistic regression analysis, clinicians were more likely to demonstrate desirable practices of exclusive breastfeeding compared to the nurses (OR 4.2; 95% CI 1.6–10.9; *p*-value = 0.003) (Table 5). Job cadre remained important even on multivariate logistic regression analysis once sex, age, facility level, cadre, and years since professional training and breastfeeding on the job training were controlled for. Clinicians (COs/AMOs/doctors) remained more likely to demonstrate desirable practice of exclusive breastfeeding than nurses (OR 3.6; 95% CI 1.2–10.8; *p*-value 0.020), in the multivariable analysis.

## Discussion

Although Tanzania is among the first countries to adopt the Innocent declaration in the 1990’s, which emphasized the importance of health worker’s role in supporting breastfeeding [25, 26], three decades after the declaration, more than half (54.5%) of respondents were not aware that their facilities had a breastfeeding policy. Our findings show better results compared to a study conducted in Indore India, which found that none of the hospitals had a breastfeeding policy that was communicated to health workers and there was no breastfeeding training [27]. Our findings suggest that the health worker’s practices are not guided and informed by the Tanzania national breastfeeding policy.

Some of the health workers had high levels of knowledge on some aspects of EBF in this study compared to other studies conducted earlier in Tanzania [28]. This improvement could be due to the influence created by peer health workers attending Prevention of Mother to Child Transmission (PMTCT) of HIV counselling training, ongoing PMTCT services at the study facilities and media coverage that promotes EBF rather than policy and guideline training.

There were incongruous results between knowledge and practice among the health workers in this study. In general health workers demonstrated a higher proportion of desirable knowledge responses (52%) than desirable practical skills (38%). This variation implies that their practice was not supported by theoretical understanding of EBF. These findings suggest that most women served by this population of health workers would not likely be adequately helped to breastfeed their infant soon after delivery. It was surprising to find that in the adjusted analysis, clinicians were almost four times more likely to have desirable practice of exclusive breastfeeding than nurses/midwives who are often involved in conducting deliveries and supporting early breastfeeding. However, clinical officer and assistant medical officers were less likely to have desirable knowledge compared to nurses (OR 0.4; 95% CI 0.1-0.09; *p*-value 0.033). Opposite findings were reported at Keffe Hospital where the doctors were found to be more knowledgeable than other health workers [19].

These findings could be possibly attributed to better clinical or practical training among clinicians compared to other health workers rather than on the job training and mentoring. The finding that the nurses who are often in contact with nursing mothers soon after delivery exhibited relatively undesirable practice is worrisome. Hospital-based health workers demonstrated more desirable results compared to those who work in dispensaries and health centres combined (OR 2.1; 95% CI 1.1–4.0; *p*-value 0.032). Of importance, on-the-job training was associated with improved knowledge after controlling for sex, age, facility level, cadre, and years since professional training and on the job breastfeeding training (OR 2.7; 95% CI 1.2–6.1; *p*-value 0.015). From these findings we might presume there were more EBF training opportunities available to the hospital based staff compared to those at the peripheral facilities. The study may also support the findings of the study conducted in Morogoro, Tanzania, which reported higher initiation (82%) of breastfeeding among women in the urban compared to those in the rural setting (52%) [25], which they attributed to higher knowledge among health workers in urban settings.

### Strengths and limitations of the study

This study is limited by the use of a convenience sample and non-validated tools and cut-off points. The strengths of this study include its purposive sampling to include several cadres of health care workers from a variety of settings, as well as urban and rural settings. The study also assessed not only knowledge but also observed practice, and assessed for associated predictive factors. This generalizability of this study is limited since we do not know whether the health workers included are representative of the population of health workers in Tanzania.

## Conclusion

The health workers at Nyamagana and Ilemela districts exhibited poorer EBF practices, compared to their knowledge. Most of the health workers had no training on EBF, as well as were not familiar with breastfeeding policy. If the EBF practice in the Tanzania is to increase from current 50% [12], health workers need to have in-depth knowledge and unequivocal practice, informed by breastfeeding policy.

## Additional file

Additional file 1: Research Questionnaire and checklist. (DOC 90 kb)

## Abbreviations

AMO: Assistant medical officer
BF: Breastfeeding
BMC: Bugando Medical Centre
CI: Confidence interval
CO: Clinical officer
CUHAS: Catholic University of Health and Allied Sciences
EBF: Exclusive breastfeeding
HIV: Human immunodeficiency virus
OR: Odds ratio
PMTCT: Prevention of mother to child transmission
TFNC: Tanzania food and nutrition centre
UNICEF: United Nations Children’s Fund
WHO: World Health Organization

## References

[1] WHO and UNICEF. Baby Friendly Hospital Initiative; Revised, Updated and Expanded for Integrated Care. Geneva: WHO Press; 2009. from http://www.who.int/nutrition//infantfeeding/9789241594950/en/index.html. Accessed 18 Feb 2012. 23926623

[2] WHO & UNICEF (1990). Innocent declaration on protection, promotion and support of breastfeeding.

[3] Huffman SL, Combest C (1990). Role of breast-feeding in the prevention and treatment of diarrhoea. J Diarrhoeal Dis Res.

[4] Cesar G, Victora J, Patrick VP, Cintia LC, Sandra MC, Fuchs SMC (1987). Evidence for protection by breastfeeding against infant deaths from infectious diseases in Brazil. Lancet.

[5] Labbok MH (2001). Effects of breastfeeding on the mother. Pediatr Clin N Am.

[6] Jones G, Steketee RW, Black RE, Bhutta ZA, Morris SS (2003). How many child deaths can we prevent this year?. Lancet.

[7] Sally I, Louisa J, Fanser DM, Cooper AM, Nolte AGW (2003). Infant feeding. Myles textbook for midwifes African edition.

[8] Newman J. How Breast Milk Protects Newborns; http://kellymom.com/pregnancy/bf-prep/how_breastmilk_protects_newborns/. Accessed 29 Feb 2016. 10.1038/scientificamerican1295-76 8525349

[9] Kramer MS, Kakuma R (2012). Optimal duration of exclusive breastfeeding. Cochrane Database Syst Rev.

[10] Poggensee G, Schulze K, Moneta I, Mbezi P, Baryomunsi C, Harms G (2004). Infant feeding practices in western Tanzania and Uganda: implications for infant feeding recommendations for HIV-infected mothers. Tropical Med Int Health.

[11] Iliff PJ, Piwoz EG, Tavengwa NV, Zunguza CD, Marinda ET, Nathoo KJ, Moulton LH, Ward JB (2005). Exclusive breastfeeding reduces the risk of postnatal HIV-1 transmission and increases HIV-free survival. AIDS.

[12] National Bureau of Statistics (NBS) Tanzania and ICF Macro (2011). Tanzania Demographic and Health Survey 2010.

[13] Aarts C, Kylberg E, Hörnell A, Hofvander Y, Gebre-Medhin M, Greiner T. How exclusive is exclusive breastfeeding? A comparison of data since birth with current status data. Section for International Maternal and Child Health, Department of Women’s and Children’s Health, Uppsala University, Sweden. 10.1093/ije/29.6.1041 11101545

[14] World Health Organization and UNICEF (2009). Global strategy for infants and young child feeding.

[15] World Health Organization. The Technical Basis and Recommendation for Action. 2nd Edition. Geneva; 1993.

[16] UNICEF (2011). State of the world children.

[17] Chaput KH, Adair CE, Nettel-Aguirre A, Musto R, Tough SC. The experience of nursing women with breastfeeding support: a qualitative inquiry. CMAJ Open. 2015. doi:10.9778/cmajo.20140113. 10.9778/cmajo.20140113 PMC4593410 26442229

[18] Tanzania Food &Nutrition Centre: A study report on infant feeding practice in context of HIV/AIDs. 2005. Final report No 2026.

[19] Okolo SN, Ogbonna C (2002). Knowledge, attitude and practice of health workers in keffi local government hospital regarding baby- friendly hospital initiatives (BFHI) practice. Eur J Clin Nutr.

[20] Mwanza Region Socio-Economic Profile. from http://www.mwanza.go.tz. Accessed 19 Dec 2011.

[21] Mwanza Region, http://en.wikipedia.org/wiki/Mwanza_Region. Accessed 19 Feb 2012.

[22] Cochran W (1963). Sampling techinque 2nd edition.

[23] UNICEF UK Baby Freindly Initiative. Breastfeeding Observation checklist. United Kingdom; 2008.

[24] WHO. Exclusive Breastfeeding and baby friendly hospital initiatives. Geneva: World Health Organization. Accessed on 18 Feb 2000 from http://www.who.int/nutrition/topics/exclusive_breastfeeding/en/.

[25] TFNC. Baby Friendly Hospital Initiatives. Dar Es Salaam: Tanzania Food and Nutrition Centre; 2005–2006. from http://www.tfnc.or.tz/eng/focus/mcn.htm. Accessed on 15 Aug 2012.

[26] Fanser DM, Cooper AM (2009). Myles textbook of midwives.

[27] Nigam R, Nigam M, Waure RR, Deshpande A, Chandork RK (2010). Breastfeeding practice in baby friendly hospital of Indore. Indian J Pediatr.

[28] Shirima R, Gebre-Medhin M, Greiner T (2001). Information and socioeconomic factors associated with early breastfeeding practices in rural and urban Morogoro, Tanzania. Acta Paediatr.

