# Supplementary material for: Predictors of knowledge and practice of exclusive breastfeeding among health workers in Mwanza city, northwest Tanzania
Source: BMC Nurs. 2016 Dec 30;15:72. doi: 10.1186/s12912-016-0192-0 (PMC5203719; doi:10.1186/s12912-016-0192-0)
Supplement: Additional file 1: — Research Questionnaire and checklist. (DOC 90 kb) [file 12912_2016_192_MOESM1_ESM.doc]

**Predictors of knowledge and practice of exclusive breastfeeding among health workers in Mwanza city, northwest Tanzania**

RESEARCH QUESTIONNAIRE

| **INTRODUCTION**  **I would like to ask you a few questions about yourself and about any professional and other training you have had** | | | | | |
| --- | --- | --- | --- | --- | --- |
|  |  | | | | **Code** |
| 1 | Name of Health Facility……………….. | | | |  |
| 2 | Health Facility Type (level)  1 = Urban Dispensary; 2 = Rural Dispensary; 3 = Urban Health Centre; 4 = Rural Health Centre; 5 = Hospital and Referral Hospital | | | |  |
| 3 | Sex  1 = Male, 2 = Female | | | |  |
| 4 | What is your age?  1 = 19 – 30 years; 2 = 31 – 40 years; 3= 41 – 50 years; 4= 51 years and above | | | |  |
| 5 | What is your cadre at this health facility?  1 = Registered Nurse/Midwife; 2 = Enrolled Nurse/Midwife; 3 = Doctor; 4 = Clinician (CO and AMO); 5 = Auxiliary Nurse/Midwife; 6 = others (specify) …….. | | | |  |
| 6 | When did you complete your latest professional training course?  1= less than 1 year; 2 = 1 to 3 years; 3 = 4 to 8 years; 4 = 9 years and more | | | |  |
| 7 | Have you ever attended any on the job training on BF (including seminars and workshop)?  1 = Yes, 2 = No | | | |  |
| 8 | If Yes, for how long were you trained……………………………… | | | |  |
| **BREASTFEEDING AND EXCLUSIVE BREASTFEEDING KNOWLEDGE**  **I would like to ask you a few questions regarding your understanding of BF and EBF in particular** | | | | | |
| 9 | Does the Health Facility were you work have a Breastfeeding Policy?  1 = Yes, 2 = No, 8 = don’t know | | | |  |
| 10 | If Yes, you are you familiar with the policy?  1 = very familiar, 2 = somewhat familiar, 3 = No t familiar | | | |  |
| 11 | Is the breastfeeding policy that serve mothers, infants and children in the health facility visibly displayed  1 = Yes; 2 = No; 9 = not applicable (if no policy) | | | |  |
| 12 | Were you trained on the breastfeeding policy?  1 = Yes; 2 = No | | | |  |
| 13 | If Yes; for how long were you trained on the breastfeeding policy  1 = Less than 5 hours; 2 = 5 to 10 hours; 3 = 11 – 18 hours; 4 = 19 hours or more; 9 = Not applicable (if not trained at all) | | | |  |
| 14 | Did the training discuss the 10 steps to successful breastfeeding?  1 = Yes; 2 = No; 8 = Don’t know; 9 = Not applicable (if not trained at all) | | | |  |
| 15 | Cumulatively how many hour of clinical mentorship after the training on breastfeeding did you undergo?  1 = Yes, for 30 minutes to 1 hour; 2 = Yes for 1 hour to 2 hours; 3 = Yes, 3 hours and more; 4 = Not at all; 9 = Not applicable if not trained | | | |  |
| 16 | Are you trained in breastfeeding counselling?  1 = Yes, during pre/in-service training; 2 = Yes, during seminar and workshop; 3 = Not all | | | |  |
| 17 | Has the facility ever received any milk formulae donations to babies over the past 1 year  1 = Yes; 2 = No; 3 = Don’t know | | | |  |
| 18 | Inspect the facility and confirm availability of the policy  1 = Has policy, posted 2 = Has policy, not posted 3 = No policy | | | |  |
| 19 | Ask the health worker to describe in her own words, what is the meaning of EBF. Using a definition confirm if the description is correct  1 = Incorrect description; 2 = partially correct; 3 = correct; 4 = very correct | | | |  |
| 20 | How soon after delivery do you think a baby should be given to the mother for skin-to-skin contact  1 = within 30 minutes; 2 = 1 to 3 hours; 3 = 4 – 6 hours; 4 = after 6 hours; 5 = not important; 8 = Don’t know | | | |  |
| 21 | How soon after delivery do you think a baby should be given to the mother must initiate breastfeeding  1 = 30 - 60 minutes; 2 = >1 to 3 hours; 3 = 6 hours; 4 = after 6 hours; 5 = not important; 8 = Don’t know | | | |  |
| 22 | For how long [in months] do you think the baby should be exclusively breastfed?  1 = less than 4 months; 2 = 4 to 6 months; 3 = more than 6 months; 8 = Don’t know | | | |  |
| 23 | Is the breast milk sufficient for babies nutrition needs even during hot weather?  1 = Yes; 2 = No; 3 = think they need some water; 8 = Don’t know | | | |  |
| 24 | Some babies cry a lot and often, do you think they need complementary [other food or drinks] before the age of 4 months  1 = strong agree; 2 = agree; 3= somewhat agree; 4 = No; 8 = Don’t know | | | |  |
| 25 | How often should the baby be breast fed?  1 = whenever hungry and on demand; 2 = at scheduled intervals; 3 = at mother’s convenience; 4 = other responses (specify --------------------------- | | | |  |
| **BREASTFEEDING PRACTICES**  **I would like to ask you some questions about helping the women breastfeed**  **(*using a check list observe step by step the health worker assisting the breastfeeding mother breastfeed her baby) – see checklist provided*** | | | | | |
| 26 | *Look at your checklist and Rank your BF observations according to the following criteria*  1 = 23 – 50 (Poor practice); 2 = 51 – 70 (Good Practice); 3 = 71 and above (Excellent practice) | | | |  |
| 27 | How do you help a mother with breastfeeding problems after delivery?  …………………………………………………………………………… | | | |  |
| 28 | Are there places or people here who could help breastfeeding mothers who need more help to breastfeed?  1 = Yes, support group; 2 = Yes, refer; 3 = No; 8 = Don’t know | | | |  |
| 29 | Do you inform pregnant women attending the clinic where you work about overcoming EBF problems?  1 = Always; 2 = sometimes; 3 = not all; | | | |  |
| **ATTITUDE**  **I would like to ask you few more questions about exclusive breastfeeding** | | | | | |
| 30 | In your own view how important is exclusive breastfeeding to infant health?  1 = very important, 2 = important; 3 = not important; 4 = not at all important. | | | |  |
| 31 | In your own views do you think colostrum is good for the new born?  1 = very good; 2 = good; 3 = Not good; 8 = Don’t know (or not sure) | | | |  |
| 32 | In your own view would you recommend bottle feeding to ‘modern’ working women?  1 = Yes always; 2 = Yes, somehow; 3 = No; 8 = Don’t know | | | |  |
| **BREASTFEEDING PRACTICE BARRIERS**  **I would like to ask you about factors affecting exclusive breastfeeding in the community and at your work-place** | | | | | |
| 33 | | In your own views, what do you think are barriers affecting EBF at community and at work place? (Prompt for each factor and get response. More than one response is expected) | | | |
|  | | Issue | 1 = I Agree | 2 = I don’t Agree | |
| A | | Limited training among health workers |  |  | |
| B | | Too many clients to provide EBF counselling |  |  | |
| C | | Lack of clear policies at health facilities regarding EBF |  |  | |
| D | | Health workers own perception and beliefs |  |  | |
| E | | Lack of knowledge among mothers |  |  | |
| F | | Misconception and/or myths in the community (*Give example of myths in this community …………………………)* |  |  | |
| G | | Lack of EBF support groups in the community |  |  | |
| H | | Promotion of milk formula and substitutes in the media, clinics, or public places |  |  | |
| I | | Inadequate [insufficient] time among working women |  |  | |
| J | | Modern life style and cultural influences in the urban setting |  |  | |

**Thank you for responding to the interview**

BREAST FEEDING PRACTICE OBSERVATION CHECKLIST

**No. of Interviewee ______________________________**

**Name of Interviewer _______________________________**

**Date of Interview __________________________________**

Observe the health worker help the mother breastfeed using the checklist below. Rank as you observe from 1 incorrect to 5 very collect. After observation, sum the column total and summarize your finding [grand total] on the shaded are as poor skill, good skill or excellent skill

| **DESCRIPTION** | **1**  **Incorrect** | **2**  **Partially Correct** | **3**  **Correct** | **4**  **Very Correct** |
| --- | --- | --- | --- | --- |
| **Mother’s position** |  |  |  |  |
| Mother relaxed and comfortable |  |  |  |  |
| Breast hanging or lying naturally |  |  |  |  |
| Easy access to nipple/areola |  |  |  |  |
| Hair /clothing do not restrict mother’s view |  |  |  |  |
| **Baby’s position** |  |  |  |  |
| Baby’s head and body in line |  |  |  |  |
| Baby held close to mother’s body |  |  |  |  |
| Baby’s whole body is supported |  |  |  |  |
| Baby’s nose opposite nipple |  |  |  |  |
| **Attachment to the breast** |  |  |  |  |
| Baby reaches or roots for the breast |  |  |  |  |
| Mother waits for baby to open mouth wide |  |  |  |  |
| Baby opens mouth wide |  |  |  |  |
| Mother brings baby swiftly towards breast |  |  |  |  |
| Baby’s chin/lower lip/tongue touches breast first |  |  |  |  |
| **During the feeding** |  |  |  |  |
| Baby’s chin touches the breast |  |  |  |  |
| Baby’s mouth wide open |  |  |  |  |
| Baby’s cheeks soft and rounded |  |  |  |  |
| Bay’s upper lip turned outwards |  |  |  |  |
| A visible, more areola seen above baby’s top lip |  |  |  |  |
| **Baby’s behavior** |  |  |  |  |
| Baby stays attached to the breast |  |  |  |  |
| Baby calm and alert at the breast |  |  |  |  |
| Slow ,deep sucking burst with pauses |  |  |  |  |
| No noise other than swallowing |  |  |  |  |
| Rhythmic swallowing seen |  |  |  |  |
| **Sub- Total (column total)** |  |  |  |  |
| **Grand Total** |  | | | |

**Max Score = 92, Minimum Score = 23**

**Interpretation: 23 – 50 Poor, 51 – 70: Good, 71 – 92: Excellent**
